# ^68^Ga-PSMA PET/CT targeted biopsy for the diagnosis of clinically significant prostate cancer compared with transrectal ultrasound guided biopsy: a prospective randomized single-centre study

**DOI:** 10.1007/s00259-020-04863-2

**Published:** 2020-07-30

**Authors:** Le-Le Zhang, Wen-Cheng Li, Zheng Xu, Nan Jiang, Shi-Ming Zang, Lu-Wei Xu, Wen-Bing Huang, Feng Wang, Hong-Bin Sun

**Affiliations:** 1 Department of Nuclear Medicine, Nanjing First Hospital, Nanjing Medical University, Nanjing, 210006 China; 2 Department of Urology, Nanjing First Hospital, Nanjing Medical University, Nanjing, 210006 China; 3 Department of Pathology, Nanjing First Hospital, Nanjing Medical University, Nanjing, 210006 China

**Keywords:** Prostate cancer, ^68^Ga-PSMA PET/CT, Targeted biopsy, Transgluteal approach

## Abstract

**Purpose:**

^68^Ga-prostate-specific membrane antigen (PSMA) positron emission tomography/computed tomography (PET/CT) is valuable for detecting primary and recurrent prostatic lesions. This study aimed to evaluate the efficacy of ^68^Ga-PSMA-11 PET/CT as a triage tool for prostate biopsy (PSMA-TB) and compare with transrectal ultrasound-guided biopsy (TRUS-GB) for the diagnosis of clinically significant prostate cancer (csPCa).

**Methods:**

This single-centre study randomly allocated 120 patients with elevated serum prostate-specific antigen (PSA) levels (> 4 ng/ml) to PSMA-PET or TRUS group. Patients with PSMA-avid lesions (SUVmax ≥ 8.0) underwent PSMA-TB via a single-puncture percutaneous transgluteal approach (*n* = 25), whilst patients with negative PSMA-PET underwent systematic TRUS-GB (*n* = 35). All patients in the TRUS group underwent TRUS-GB directly (*n* = 60).

**Results:**

PCa and csPCa were detected in 26/60 (43.3%) and 24/60 (40.0%) patients in the PSMA-PET group and 19/60 (31.6%) and 15/60 (25.0%) in the TRUS group, respectively. In the PSMA-PET group, the detection rate of PCa and csPCa were significantly higher in PSMA-PET-positive than negative patients (PCa, 23/25 (92.0%) vs 3/35 (8.6%), *P* < 0.01; csPCa, 22/25 (88.0%) vs 2/35 (5.7%), *P* < 0.01). PSMA-TB detected significantly more PCa and csPCa than TRUS-GB in the TRUS controls (PCa, 21/25 (84.0%) vs 19/60 (31.6%), *P < 0.01*; csPCa, 20/25 (80.0%) vs 15/60 (25.0%), *P* < 0.01). PSMA-PET detected significantly more cases of csPCa amongst patients with PSA 4.0–20.0 ng/ml than TRUS (27.02% vs 8.82%, *P* < 0.05). No haematuria, urinary retention or pelvic infection was observed after PSMA-TB compare with TRUS-GB.

**Conclusions:**

^68^Ga-PSMA-11 PET/CT is a feasible imaging technique that may serve as a triage tool for prostate biopsy, and may improve the detection rate of csPCa compared with TRUS-GB, especially in patients with serum PSA 4.0–20.0 ng/ml.

## Introduction

Systematic transrectal ultrasound-guided biopsy (TRUS-GB) is currently the main technique for the diagnosis of prostate cancer (PCa). However, this random, non-targeted approach increases the detection of low-risk disease, whilst about 18% clinically significant cancers (csPCa) are missed [1, 2]. Multi-parametric nuclear magnetic resonance (mpMRI) and TRUS fusion targeted biopsy (TB) improved the detection rate of csPCa [3–5]. However, small foci and disease within the central gland may not be easily identified by mpMRI, and up to 35% of csPCa may be invisible to mpMRI [6], thus decreasing the diagnostic efficacy of MRI/TRUS-TB.

Prostate-specific membrane antigen (PSMA) is highly expressed in most primary and metastatic castration resistant PCa [7, 8], which is a highly specific prostatic epithelial cell transmembrane protein and an ideal molecular target for PCa. PSMA inhibitors conjugated with the radionuclides ^68^Ga and ^18^F-fluoride have been well-explored and successfully translated for the clinical diagnosis of PCa in the last decade [9, 10]. ^68^Ga-PSMA positron emission tomography/computed tomography (PET/CT) is a valuable method for detecting biochemical recurrence and malignant lymph nodes [11]. A recent study demonstrated that ^68^Ga-PSMA PET/CT also showed high sensitivity for identifying nodal and/or distant metastases compared with TRUS, and was thus a useful tool for tumour staging [12]. Moreover, tumour uptake, which represents PSMA expression, was correlated with a Gleason score in the primary prostatic tumour [13]. However, a limited number of studies have focused on the primary prostatic lesion [14, 15]. We previously showed that ^68^Ga-PSMA PET/CT had higher sensitivity for the detection of lymphadenopathy and visceral metastasis compared with mpMRI, and described the clinical characteristics of intra-prostatic primary lesions including tumour size, shape, and location [16–19].

The ideal prostate biopsy strategy would improve detection rate of csPCa and minimize the detection of indolent disease, thus avoiding the over-treatment of patients with PCa. ^68^Ga-PSMA PET/CT has shown to be more sensitive for the detection of primary prostatic lesions and regional lymphadenopathy compared with TRUS and MRI [17, 18]. We therefore hypothesized that ^68^Ga-PSMA PET/CT could serve as a triage tool for prostate biopsy. This study aimed to investigate the feasibility of ^68^Ga-PSMA PET/CT-targeted biopsy (PSMA-TB) for detecting PCa, especially csPCa.

## Patients and methods

### Patients and study design

**Fig. 1:**
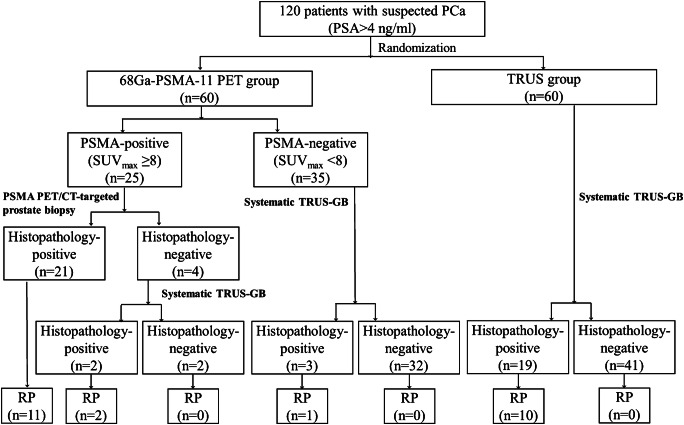
CONSORT diagram for this study

This study was approved by the Nanjing First Hospital, and all patients signed an informed consent. A total of 120 patients with elevated serum prostate-specific antigen (PSA) levels (> 4.0 ng/ml) were consecutively enrolled and randomized into PSMA-PET and TRUS groups. Each patient was assigned a random computer-generated number, and patients with odd numbers were assigned to the PSMA-PET group and patients with even numbers to the TRUS group. All patients in the PSMA-PET group (*n* = 60) underwent ^68^Ga-PSMA-11 PET/CT. PSMA-TB was subsequently performed if PSMA PET/CT was highly suggestive of PCa, and systematic TRUS-GB was performed if PSMA PET/CT was negative. For patients with multiple intra-prostatic PSMA-avid lesions, the lesion with the highest uptake was selected as the puncture target. If PSMA-TB were negative, systematic TRUS-GB plus two cores of suspicious-lesion biopsies were performed within 2 days. All patients in the TRUS group (*n* = 60) underwent direct systematic TRUS-GB. Clinically significant PCa was defined as any Gleason score ≥ 7 (3 + 4) and a lesion diameter > 0.5 cm^3^, or T3/T4 clinical stage. The CONSORT diagram for this study is shown in Fig. 1.

### ^68^Ga-PSMA PET/CT

The precursor PSMA-HBED (DKFZ-PSMA-11; GMP-compliant grade) was obtained from ABX advanced biochemical compounds GmbH (Germany). ^68^Ga was obtained from a ^68^Ge/^68^Ga generator (ITM Company, Germany). ^68^Ga-PSMA-11 was radiolabelled using an automated module (ITM). All products were prepared using good manufacturing practice and were non-pyrogenic and sterile. Radiochemical purity and stability were determined by analytical reverse phase high-performance liquid chromatography. The radiochemical purity of all products administered to patients for imaging was > 99%. PET/CT was performed (uMI780, United Imaging, China) 45–60 min after injection of 111–185 × 10^6^ MBq ^68^Ga-PSMA-11 (3–5 mCi). CT images were used for attenuation correction and accurate localization. PET imaging was acquired immediately after CT scanning (matrix 256) with a 15.5 cm field of view, with 3 min acquisition for each bed position. Pelvic imaging in the prone position was specifically acquired for prostate TB. Reconstruction was conducted using an ordered subset expectation maximization algorithm with four iterations/eight subsets, and Gauss-filtered to an in-plane spatial resolution of 3 mm at full-width at half-maximum. PET/CT fusion was performed using a uMI 780 workstation. All lesions were displayed in three planes (transaxial, coronal and sagittal), and the region of interest (ROI) was delineated from the PET/CT fusion image.

### Image interpretation

Images were examined by one radiologist and one nuclear medicine physician who were blinded to the clinical characteristics and pathology. Visual and semi-quantitative analyses were used to detect the primary lesion and lymph node metastasis. Region of interest (ROI) was drawn around the primary prostatic lesion with 40% maximal standardized uptake values (SUVmax) cut-off in the 1 h postinjection fusion image, SUVmax of the prostatic primary lesions and metastasis were acquired from the ROI. In this study, lesions with abnormal focal uptake in the prostate gland, tracer activity higher than the surrounding background and a SUVmax higher than the cut-off value of 8.0 were defined as intra-prostatic primary lesions, and suspected with csPCa based on our previous data and other clinical studies [13, 20]. Considering the lymph node drainage pattern of prostate cancer, any pelvic lymph node with focal increased uptake was regarded as a metastatic lymph node. For csPCa detection, a PSMA-avid prostatic lesion and SUVmax ≥ 8.0 were considered necessary prerequisites for transgluteal PSMA-TB. Lesion size, position and number in the prostate bed were identified by a urologist, radiologist and nuclear medicine physician on PET/CT. The *volume* of each lesion was calculated using the equation: lesion volume = *1/2* × *A* × B2 (mm^3^) where A and B represent the long and short diameters of the lesion, respectively. For multiple positive lesions, the lesion with the highest SUVmax, which may present with the highest aggressiveness, was selected as the puncture target. Patients with lower uptake in the primary prostatic lesion (SUVmax < 8.0) underwent systematic TRUS-GB.

### Transgluteal PSMA PET/CT-TB

**Fig. 2:**
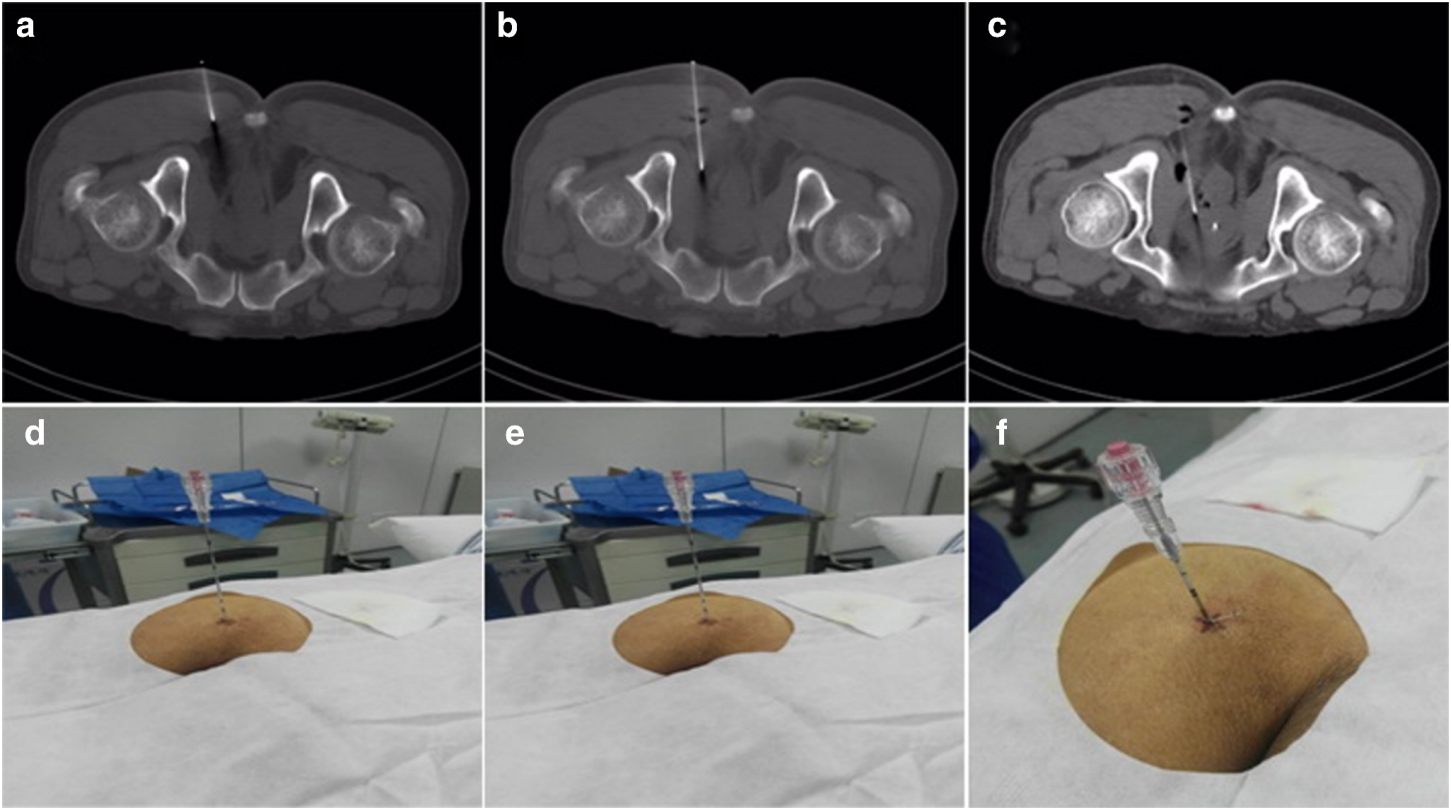
^68^Ga-PSMA-TB was performed in the prone position, using a single puncture percutaneous transgluteal approach technique. The needle was introduced into the gluteus maximus (**a** and **d**), the ischial anal fossa (**b** and **e**) and intra-prostatic lesion (**c** and **f**) under CT guidance in real-time

All patients signed consent for this novel targeted biopsy procedure. Enemas and prophylactic antibiotics were not required. Aspirin and other anticoagulants were withdrawn for 7 days prior to the procedure. Pelvic CT was performed in the prone position, and the optimal cognitive fusion of PET and CT images was conducted. Once the index lesion in the prostate was determined, the puncture path, angle and depth were pre-simulated on the CT image. Metal palisade labelling was used to locate the puncture point on the patient’s buttock, and a single-puncture percutaneous transgluteal approach was implemented with 1% lidocaine for local anaesthesia. Under CT guidance, an Argon Angiotech 17G biopsy trocar (Athens, TX, USA) was introduced through the gluteus maximus, the ischial anal fossa, and the anal elevator muscle and positioned cognitively at the target point (Fig. 2). Two to four biopsy specimens were taken in the target area using the co-axial needle technique. Tissue samples were analysed by a pathologist. A post-biopsy pelvic CT (5–10 min after puncture) was performed to exclude immediate complications such as active pelvic bleeding or rectal bleeding. The procedure was well-tolerated, with an overall procedure time of approximately 20 min.

### Systematic TRUS-GB

Patients underwent a standard systematic TRUS-GB protocol. Briefly, oral ciprofloxacin was routinely administered for 3 days, and an enema was performed 1 h before the procedure. Patients were placed in the left lateral position and local anaesthesia was applied to the anus. Systematic prostate biopsy was performed using an 18G needle (Bard, Covington, GA, USA) under B ultrasound guidance (Hitachi Noblus, Tokyo, Japan). A total of 12 core aspirations were performed in four sagittal planes of the medial line of the prostate lobe on both sides and the lateral side of the peripheral belt.

### Histopathology

Biopsy specimens and the tumour cell ratio of the whole tissue sample were analysed by a pathologist with 5 years’ experience in urinary pathology. Histopathology positive biopsy specimens were compared with gross tissue histopathology in patients who underwent radical prostatectomy. The final pathology for each patient was confirmed as follows: (1) gross tissue histopathology after radical prostatectomy; (2) biopsy histopathology if patients did not undergo radical prostatectomy; and (3) biopsy specimen histopathology of TRUS-GB plus two core suspicious lesions if patients were negative for PSMA-TB.

### Data analysis

Statistical analysis was performed using SPSS, version 24.0 (Chicago, IL, USA). The normality of the data was tested by Kolmogorov–Smirnov analysis. Continuous variables with a normal distribution were presented as mean (± standard deviation, SD) and analysed by Student’s *t* tests. Continuous variables with a non-normal distribution were presented as median (interquartile range) and analysed by Mann–Whitney *U* tests. *χ*^2^ or Fisher’s exact tests were used to compare the differences between categorical variables. A *P* value < 0.05 was considered statistically significant.

## Results

### Patient demographics

The clinical data are shown in Table 1. Age, prostate volume and PSA level were comparable between the PSMA-PET and TRUS groups.

**Table 1:** Clinical characteristics of 120 patients enrolled in the study

Clinical characteristics	Total	PET group (*N* = 60)	TRUS group (*N* = 60)	*P*
Age/y	71.1 ± 8.4	71.6 ± 9.1	70.6 ± 7.7	0.5204
Prostate volume/ml PSA(ng/ml)	64.1 ± 33.6 28.2 ± 26.5	62.9 ± 29.1 27.4 ± 28.1	65.4 ± 38.9 28.5 ± 27.9	0.4909 0.9977
PSA Subgroups				0.3678
4~20 ng/ml	71	37	34	
20 ~ ng/ml	49	23	26	
No. of PCa(%)	45(37.5)	26(43.3)	19(31.6)	0.1869
No. of csPCa(%)	39(32.5)	24(40.0)	15(25.0)	0.0794

*PCa*, prostate cancer; *csPCa*, clinically significant prostate cancer

### PCa detection between PSMA TB and TRUS-GB

The overall detection rates of PCa and csPCa were 37.5% (45/120) and 32.5% (39/120), respectively. PSMA-PET detected 43.3% (26/60) PCa and 40.0% (24/60) of csPCa, whilst TRUS detected 31.6% (19/60) PCa and 25.0% (15/60) of csPCa. There was no significant difference in detection rates for PCa (χ^2^ = 1.74, *P* > 0.05) and csPCa (χ^2^ = 3.08, *P* > 0.05).

**Fig. 3:**
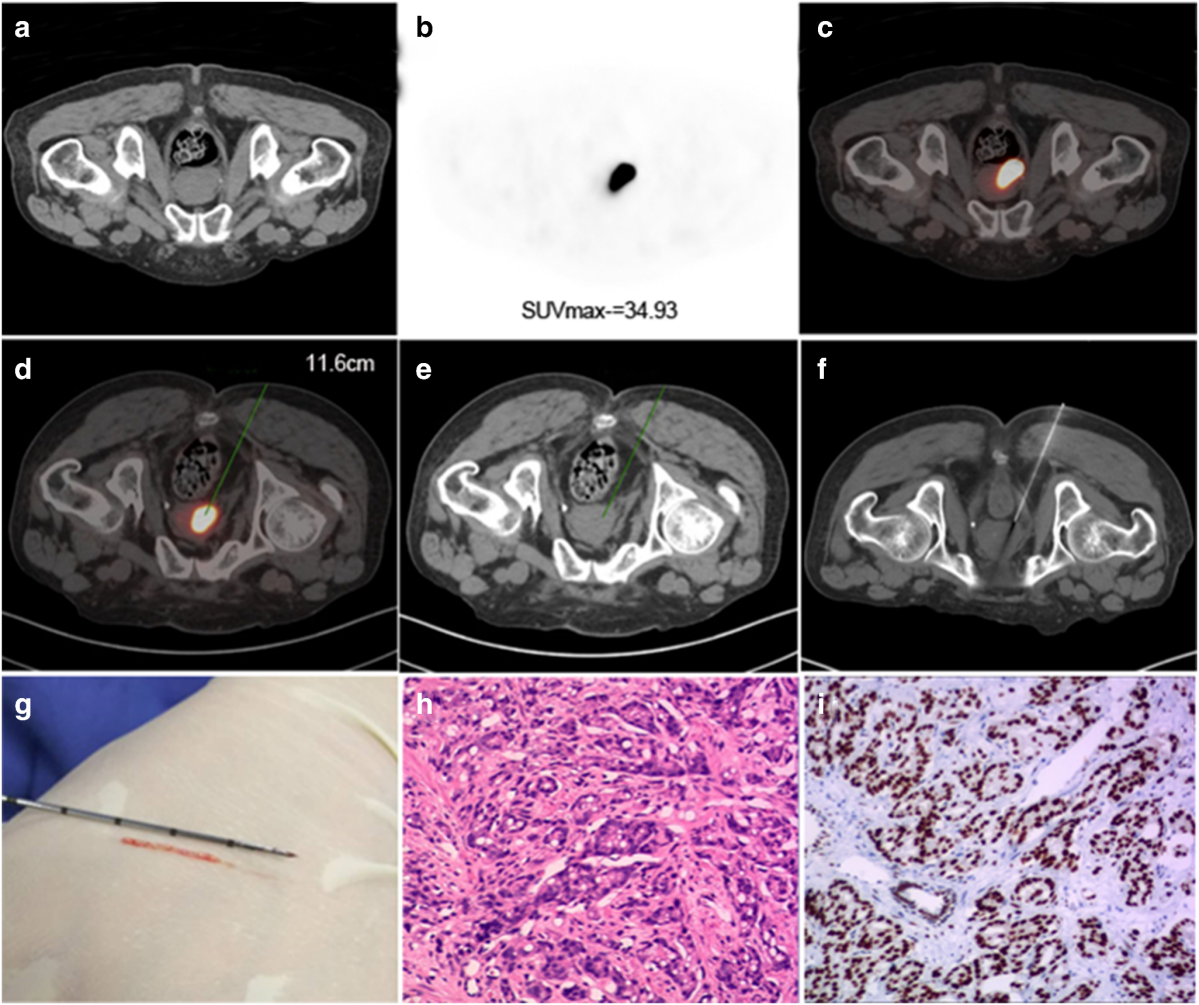
PSMA-TB was performed in an 85-year-old man with serum PSA 32.67 μg/ml. **a** CT image; **b** PSMA-avid lesion (SUVmax 34.93); and **c** fusion image in the prone position. Puncture path and angle pre-simulated on **d** fusion image and **e** CT image in the same scanning plane with a puncture depth of 11.6 cm. **f** The needle was guided into the target lesion via a transgluteal approach on CT image. **g** Biopsy specimen (1.8 cm long). **h** The lesion specimen was positive for PCa (haematoxylin and eosin staining, 10 × 10) with Gleason score 7 (4 + 3). **i** Positive PSMA expression was confirmed by EnVision immunostaining

Amongst 25 patients with PSMA-avid lesions (SUVmax 22.5 ± 14.72), 23 were true positive, of whom 21 were diagnosed by a single needle puncture (including two patients with negative TRUS-GB prior to this study). Two patients were initially negative on PSMA-TB but confirmed by supplementary TRUS-GB, and two patients who received both PSMA-TB and TRUS-GB followed by transurethral resection of the prostate gland were true negative, with a final diagnosis confirmed as benign disease. In the PSMA-PET group, the detection rates of PCa and csPCa were significantly higher in PSMA-PET-positive compared with PSMA-PET-negative patients (23/25, 92.0% vs 3/35, 8.6%, χ^2^ = 41.34, *P* < 0.01 and 22/25, 88.0% vs 2/35, 5.7%, χ^2^ = 42.68, *P* < 0.01, respectively) (Fig. 3, Table 2).

**Table 2:** Clinical characteristics of PSMA-TB in 25 patients

	Age (y)	PSA(ng/ml)	SUVmax PSMA-PET	Number of lesions	Lesion-size Short*Long (cm) PSMA-PET	PSMA-TB (Gleason)	Cancer tissue ratio (%)	TRUS-GB (Gleason)	Pathological outcome after RP (Gleason)
1	78	100.00	29.32	1	2.88*3.78	PCa, 3 + 4 = 7	80		RP, PCa3 + 4 = 7
2	64	18.54	13.99	1	1.15*1.93	PCa, 3 + 4 = 7	35	BPH	RP, PCa4 + 3 = 7
3	85	8.85	11.48	1	0.88*0.97	PCa, 3 + 4 = 7	50		N
4	89	14.76	20.52	1	1.15*2.21	PCa, 3 + 3 = 6	20	BPH	N
5	64	129.50	28.16	3	0.98*1.50	PCa, 4 + 4 = 8	80		RP, PCa4 + 4 = 8
6	70	52.05	15.20	1	1.10*1.50	PCa, 4 + 4 = 8	80		RP, PCa4 + 4 = 8
7	82	39.00	11.56	2	0.70*1.17	PCa, 4 + 3 = 7	20		RP, PCa4 + 4 = 8
8	75	15.26	11.91	1	0.59*0.74	PCa, 4 + 3 = 7	70		RP, PCa4 + 3 = 7
9	57	43.91	22.45	2	1.07*1.50	PCa, 4 + 3 = 7	50		RP, PCa4 + 3 = 7
10	85	32.67	34.93	1	2.16*3.06	PCa, 4 + 4 = 8	100		N
11	80	12.98	9.97	2	0.63*1.26	BPH		PCa, 3 + 4 = 7	RP, PCa3 + 4 = 7
12	68	36.24	12.90	1	0.52*0.64	BPH		PCa,4 + 3 = 7	RP, PCa4 + 3 = 7
13	63	98.86	30.55	2	2.00*3.20	PCa, 4 + 3 = 7	90		N
14	55	9.899	30.99	1	0.78*1.48	PCa, 4 + 4 = 8	20		N
15	85	15.37	10.18	2	0.88*1.20	PCa, 3 + 4 = 7	30		N
16	65	6.31	13.46	1	0.49*1.05	PCa, 4 + 4 = 8	50		RP, PCa4 + 4 = 8
17	78	79.60	60.69	3	3.60*5.90	PCa, 4 + 4 = 8	50		RP, PCa4 + 4 = 8
18	90	17.98	20.65	2	0.93*1.78	PCa, 4 + 4 = 8	50		N
19	73	35.60	8.73	1	1.64*2.36	PCa, 4 + 3 = 7	100		N,APR
20	72	31.00	62.40	1	2.11*2.86	PCa, 4 + 3 = 7	90		RP,PCa4 + 4 = 8
21	83	30.97	17.99	2	1.41*1.83	PCa, 3 + 4 = 7	50		N
22	75	96.04	44.90	2	1.93*4.15	PCa, 4 + 4 = 8	70		N
23	75	38.00	19.40	2	0.79*1.37	PCa, 4 + 5 = 9	50		RP, PCa4 + 5 = 9
24	76	57.67	9.63	1	0.32*0.47	BPH		BPH	TURP
25	74	42.82	10.75	2	0.33*0.66	BPH		BPH	TURP

*RP*, radical prostatectomy; *BPH*, benign prostate hyperplasia; *N*, no operation; *APR*, abdomino-perineal resection with rectal cancer prior to prostate biopsy; *TURP*, transurethral resection of the prostate

**Fig. 4:**
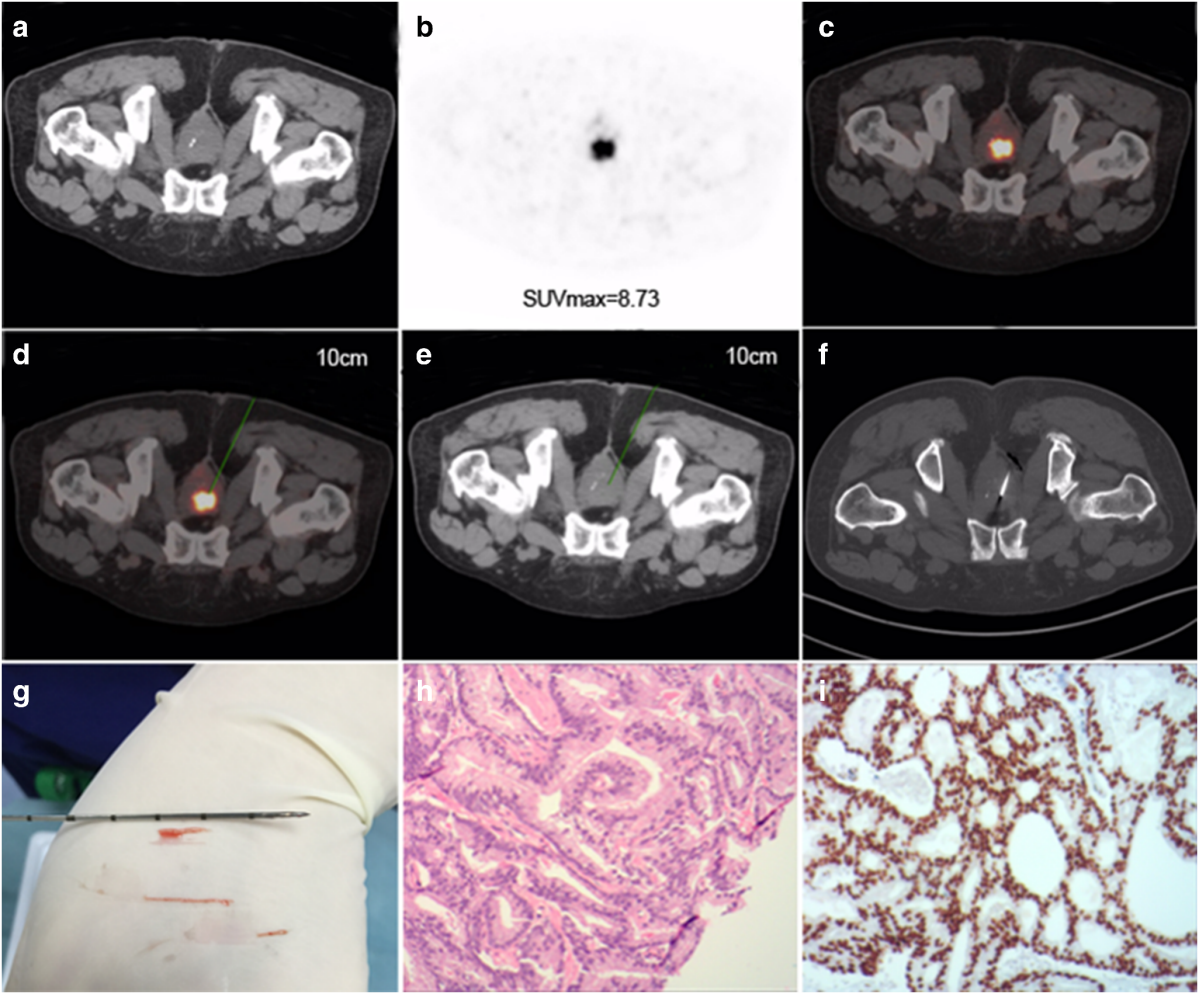
PSMA-TB was performed in a 73-year-old man (without rectal access) with serum PSA 35.6 μg/ml who had undergone rectal surgery for rectal carcinoma before enrolment in the study. **a** Normal CT; **b** PSMA-avid lesion (SUVmax 8.73); and **c** fusion image. Puncture path and angle pre-simulated on **d** fusion image and **e** CT image in the same scanning plane with a puncture depth of 10.0 cm. **f** CT image of transgluteal biopsy needle guided into the target lesion. **g** Three specimens were taken using the co-axial needle technique with lengths of 0.8, 1.0 and 2.0 cm. **h** Specimen was positive for PCa (haematoxylin and eosin staining, 10 × 10) with Gleason score 7 (4 + 3). **i** Positive PSMA expression was confirmed by EnVision immunostaining

PSMA-TB detected PCa in 21/25 (84.0%) and csPCa in 20/25 (80.0%) patients with PSMA-avid lesions, whereas TRUS-GB detected PCa in 19/60 (31.6%) (χ^2^ = 19.4, *P* < 0.01) and csPCa in 15/60 (25.0%) (χ^2^ = 22.0, *P* < 0.01) TRUS control patients. In one patient with previous abdomino-perineal resection due to rectal cancer (without rectal access), PCa (Gleason 4 + 3) was finally confirmed by this novel puncture technique (Fig. 4).

### Diagnostic efficacy in patients with different serum levels of PSA

Amongst patients with PSA 4.0–20.0 ng/ml, the detection rate of csPCa was significantly higher in the PSMA-PET (10/37, 27.02%) than that of TRUS group (3/34, 8.82%) (χ^2^ = 3.93, *P* < 0.05). However, no significant difference existed in csPCa detection between PSMA-PET and TRUS in patients with PSA > 20.0 ng/ml (60.9%, 14/23 vs 46.2%, 12/26, χ^2^ = 1.06, *P* > 0.05) (Table 3).

**Table 3:** Diagnostic efficacies of csPCa detection by PSMA-TB and TRUS-GB in patients with different serum levels of PSA

PSA subgroups		Patients	csPCa(n)	χ^2^	*P*
4 ~ 20 ng/ml				3.93	< 0.05
PET Group	(+)	9	8		
	(−)	28	2		
TRUS Group		34	3		
20 ~ ng/ml				1.06	> 0.05
PET Group	(+)	16	14		
	(−)	7	0		
TRUS Group		26	12		

### Tumour samples and histopathology

Amongst 25 patients who underwent PSMA-TB, 13 with PCa were confirmed by gross tissue histopathology after radical prostatectomy, and 10 patients with PCa confirmed by biopsy histopathology. Gleason score was underestimated by biopsy histopathology in three patients, and adjusted from 3 + 4 to 4 + 3 in one and from 4 + 3 to 4 + 4 in two patients after radical prostatectomy.

In the 21 of 25 cases of PCa diagnosed by single needle puncture, the proportions of cancer tissues were 100% in two, 90% in two, 80% in three, 70% in two, 50% in seven, and < 50% in five cases.

### Lesion size and relation with histopathology

We assessed the relationship between histopathology and lesion size in the 25 patients with PSMA-avid lesions (volume, long diameter, short diameter and long/short diameter ratio). There was no significant differences in lesion size between patients with higher Gleason scores (≥ 7) and lower Gleason scores (< 7) (all *P* > 0.05) (Table 4).

**Table 4:** Sizes of PCa lesions with different Gleason scores

Variable	Gleason score < 7	Gleason score > =7	*P*
Volume	1.07 (0.34, 2.16)	0.77 (0.44, 6.75)	> 0.05
Long	1.67 (1.14, 2.25)	1.50 (1.43, 2.96)	> 0.05
Short	1.11 (0.82, 1.47)	0.98 (0.79, 2.02)	> 0.05
L/S	1.38 (1.29, 1.62)	1.67 (1.47, 1.91)	> 0.05

### Adverse events associated with PSMA-TB

Compared with systematic TRUS-GB, the needle did not pass via the rectum for PSMA-TB and no bowel preparation, including preoperative enema and pre-antibiotics, was therefore needed. The total time for PSMA-TB was thus only 20–30 min, and no postoperative antibiotics were administered during and after puncture. PSMA-TB was well-tolerated, with only one patient experiencing a few episodes of haematuria after the TB. However, haematuria occurred in 10 patients, urine retention in three patients and rectal infection in one patient after TRUS-GB.

## Discussion

^68^Ga-PSMA-11 PET/CT has been well-documented for the early detection of biochemical recurrence of PCa, even in patients with low PSA levels [10]. Recent data that further validated PSMA PET had some merits in the preoperative staging of primary PCa, higher ^68^Ga-PSMA uptake correlated with higher Gleason score and higher aggressiveness [11, 17]. ^68^Ga-PSMA PET is of great value for detecting index lesions and high-risk disease, with significant impact on the clinical management of PCa [21, 22]. TRUS-GB is widely used for the diagnosis of PCa; however, this non-targeted biopsy technique was associated with some adverse events and missed some csPCa [23, 24]. A recent systematic review reported that MRI fusion biopsy improved the detection rate of csPCa compared with TRUS-GB [25]. However, mpMRI usually misses some lesions located in the transition and central zones, and the limited specificity of MRI for detecting PCa also decreases its diagnostic efficacy as a triage tool for biopsy. PSMA-TB has been reported in limited case studies [26, 27], including one in which a patient with four negative MRIs and six negative TRUS-GBs was finally diagnosed with PCa (Gleason 3 + 4) by PSMA-TB. To the best of our knowledge, the current study is the first to address the feasibility of ^68^Ga-PSMA PET as a triage tool for prostate biopsy.

In this pilot study, we compared the difference in detection rates of PCa between PSMA-TB and TRUS-GB and developed a novel transgluteal PSMA-TB technique, which was easy to perform in the prone position. The needle was inserted transgluteally rather than transrectally, thus avoiding complications such as rectal bleeding and infection. Except for one patient who experienced a few episodes of haematuria, there were no other complications during or after the puncture procedure. This novel approach allows the puncture needle to be adjusted in real-time by CT guidance to ensure that it is located close to the inside of the anal levator muscle, thereby avoiding damage to the pudendal vasculature and pudendal nerves. We obtained two to four biopsy specimens with only one entry site using the co-axial needle technique. Biopsy histopathology confirmed the feasibility of this novel PSMA-TB. The proportion of cancer tissues in the whole tissue sample was > 50% in 16 patients and < 50% in five cases. The Gleason score in three patients was underestimated by PSMA-TB and adjusted after radical prostatectomy.

For patients with multiple intra-prostatic lesions, the lesion with the highest SUVmax was referred to as the index lesion and selected as the puncture target. The size and position of the lesions are also important parameters, and large lesions are easier to biopsy. In this study, SUVmax cut-off value of 8.0 was determined as the csPCa threshold. Amongst 23 cases of PCa, 22 were finally confirmed as csPCa (SUVmax 8.73–40.69), and the detection rate of csPCa was 88.0%, with a sensitivity and specificity of 91.7% and 94.1%, respectively. Fendler et al. [14] reported that an SUVmax cut-off of 6.5 for PCa diagnosis resulted in a sensitivity of 67% and specificity of 92%. SUVmax reflects the tumour expression of PSMA, with higher grade tumours (Gleason score > 7) usually associated with much higher SUVmax values ranging from 16 to 21, compared with intermediate and lower grade tumours with SUVmax values of 8.2–8.8 and 5.9–9.6, respectively. SUVmax < 3.1–6.5 is strongly suggestive of benign disease [13, 20]. In this study, intra-prostatic lesions showed scattered tracer uptake that was not significantly higher than that of the prostate gland, and only three patients (8.6%, 3/35) were confirmed with PCa (SUVmax 6.36–6.76) by TRUS-GB, including two cases of csPCa (SUVmax 6.71–6.76). It has been reported that about 50% of patients missed by PSMA-PET/CT had low-grade PCa (Gleason 3 + 3) or a tumour burden < 25% [14, 28]. We therefore suggest active monitoring rather than excessive TRUS-GB for patients with elevated serum PSA but no focal uptake on PSMA PET/CT (SUVmax < 8.0). However, further studies are needed to validate this hypothesis.

The efficiency of PSMA-TB was further evaluated in patients with different levels of serum PSA. For patients with PSA 4.0–20.0 ng/ml, the detection rate of csPCa was significantly higher in the PSMA-PET (27.02%) compared with the TRUS group (8.82%), whilst the efficacies of the two procedures were comparable in patients with PSA > 20.0 ng/ml. These findings suggest that PSMA-TB might be a better option in patients with low PSA levels (< 20.0 ng/ml), leading to an improved detection rate of csPCa. However, PSMA-TB had a better detection rate for PCa and fewer adverse events in patients with serum PSA > 20.0 ng/ml or large PSMA-avid index lesions. Urinary retention, pelvic infection and lower limb pain or numbness are common side effects of TRUS-TB. However, PSMA-TB was associated with fewer adverse effects in this study, and patients therefore preferred PSMA-TB to TRUS-TB. PSMA-TB thus demonstrated good clinical potential compared with TRUS-GB. In summary, ^68^Ga-PSMA PET/CT has great merits for the detection of csPCa compared with TRUS and biopsy, whilst PSMA-TB is a novel technique with higher detection rate for csPCa but fewer adverse events, and might thus be an option for patients with PSMA-avid lesions. A single-puncture percutaneous transgluteal approach was shown to be feasible, well-tolerated and easy to perform.

The current single-centre study evaluated the feasibility of ^68^Ga-PSMA PET/CT as a triage tool for the diagnosis of PCa. However, this study had some limitations, first, mpMRI was not routinely used and the diagnostic efficacy was not compared with MRI-guided biopsy for economic reasons. Second, patients with elevated serum PSA were randomly enrolled in the study, including some with benign prostatic disease, which decreased the sensitivity of ^68^Ga-PSMA PET/CT. Third, biopsy was performed using a single-puncture technique, which might have underestimated the tumour burden. Due to variable heterogeneity of PCa, multiple internal prostatic lesions are usually observed, and selection of the PSMA-avid lesion as the target might miss low-PSMA-expressing lesions. Tumours with negative or mild PSMA expression, which is not an indication for PSMA-TB, might be missed by PSMA-PET [29].

In conclusion, ^68^Ga-PSMA PET/CT may serve as a triage tool for prostate biopsy, and PSMA-TB has higher sensitivity for the detection of csPCa than TRUS-GB. A novel percutaneous transgluteal approach involving a single puncture might improve the detection rate of csPCa. This minimally invasive targeted biopsy technique has great potential for the precise diagnosis of PCa.
